# *Staphylococcus aureus* from 152 cases of bovine, ovine and caprine mastitis investigated by Multiple-locus variable number of tandem repeat analysis (MLVA)

**DOI:** 10.1186/s13567-014-0097-4

**Published:** 2014-10-02

**Authors:** Dominique Bergonier, Daniel Sobral, Andrea T Feßler, Eric Jacquet, Florence B Gilbert, Stefan Schwarz, Michaël Treilles, Philippe Bouloc, Christine Pourcel, Gilles Vergnaud

**Affiliations:** INRA, UMR1225, IHAP, 31076 Toulouse, France; Université de Toulouse, INP, ENVT, UMR1225, IHAP, 31076 Toulouse, France; UMT INRA-ENVT-Institut de l’Élevage “Small Ruminants Health Management”, 31076 Toulouse, France; Université Paris-Sud, Institut de Génétique et Microbiologie, UMR 8621, 91400 Orsay, France; CNRS, Orsay, France; Ceeram (Centre Européen d’Expertise et de Recherche sur les Agents Microbiens), 44240 La Chapelle sur Erdre, France; Institute of Farm Animal Genetics, Friedrich-Loeffler-Institute, 31535 Neustadt-Mariensee, Germany; ICSN, CNRS, UPR2301, IMAGIF qPCR-Platform, 91198 Gif-sur-Yvette, France; INRA, UMR1282, ISP, 37380 Nouzilly, France; Laboratoire départemental d’analyses de la Manche (LDA50), 50000 Saint-Lô, France; ENSTA ParisTech, 91762 Palaiseau, France

## Abstract

*Staphylococcus aureus* is one of the main etiological agents of mastitis in ruminants. In the present retrospective study, we evaluated the potential interest of a previously described automated multiple loci Variable Number of Tandem Repeats (VNTR) Assay (MLVA) comprising 16 loci as a first line tool to investigate the population structure of *S. aureus* from mastitis. We determined the genetic diversity of *S. aureus* strains from cases of clinical and subclinical mastitis in dairy cattle (*n* = 118, of which 16 were methicillin-resistant), sheep (*n* = 18) and goats (*n* = 16). The 152 strains could be subdivided into 115 MLVA genotypes (including 14 genotypes for the ovine strains and 15 genotypes for the caprine strains). This corresponds to a discriminatory index (*D*) value of 0.9936. Comparison with published MLVA data obtained using the same protocol applied to strains from diverse human and animal origins revealed a low number (8.5%) of human-related MLVA genotypes among the present collection. Eighteen percent of the *S. aureus* mastitis collection belonged to clonal complexes apparently not associated with other pathological conditions. Some of them displayed a relatively low level of diversity in agreement with a restricted ecological niche. These findings provide arguments suggesting that specific *S. aureus* lineages particularly adapted to ruminant mammary glands have emerged and that MLVA is a convenient tool to provide a broad overview of the population, owing to the availability via internet of databases compiling published MLVA genotypes.

**Electronic supplementary material:**

The online version of this article (doi:10.1186/s13567-014-0097-4) contains supplementary material, which is available to authorized users.

## Introduction

Although several bacterial pathogens can cause mastitis, *Staphylococcus aureus* is one of the most prevalent etiologic agents of this disease in dairy cattle [1], and the most important in terms of frequency and clinical severity in goats and sheep [2,3]. As an agent of intra-mammary infections, this pathogen can contaminate the bulk milk tank and thus may constitute a bacteriological hazard for raw milk dairy products consumed. In this context, molecular subtyping tools are of great interest for the comparison of genotypes in order to identify sources and transmission routes for control improvement.

During the past decade, the epidemiology of *S. aureus* mastitis in dairy cattle has been studied using various molecular typing methods. Techniques that rely on the comparison of electrophoretic patterns, such as Pulsed-Field Gel Electrophoresis (PFGE) [4-6], Random Amplification of Polymorphic DNA (RAPD) analysis [7], ribotyping [5,8] and Multi-Locus Enzyme Electrophoresis (MLEE) [9] proved to be highly discriminatory. Nevertheless the comparison between laboratories of pattern-producing assays is difficult from the data quantification and sharing points of view since they require the implementation of very strict protocols. Sequence-based typing systems such as Multiple Locus Sequence Typing (MLST) or *spa* typing overcome these problems by producing sharable and easily storable numeric-format results [10-13]. MLST is based on partial sequencing of seven housekeeping genes. *spa* typing is based on sequencing of a highly polymorphic tandem repeat locus showing internal variations of repeat units. When applied to *S. aureus*, MLST has a low discriminatory power for a relatively high cost, so that some investigators are evaluating the practical feasibility of replacing MLST by whole genome sequence analysis [14,15]. *spa* locus typing alone is not always a robust indicator of genetic background, as illustrated for instance in an ST398 investigation [16] and is often used in combination with MLST [17]. During the last few years, different Multiple Loci VNTR (Variable Number of Tandem Repeats) Analysis (MLVA) schemes were developed for *S. aureus* subtyping [18-20] and represent a promising alternative or complement to MLST and *spa*. In addition to its much higher discriminatory power as compared to previous techniques, MLVA was able to correctly predict clonal complex (CC) assignment and consequently benefit from the strong phylogenetic content provided by MLST analysis [19]. Automated capillary-based MLVA assays for *S. aureus* genotyping using 8 or 16 VNTR loci have been published [21,22]. The second assay, called MLVA16_Orsay_ was demonstrated as being highly suitable for genotyping *S. aureus* isolates from human, animal and food sources [22].

In recent years several MLST-based studies investigating mastitis [6,13,23] have shown the existence of an important host-specificity of *S. aureus* strains. These studies described CC including *S. aureus* mainly isolated from humans (CC7, CC8, CC22, CC25, CC30, CC45 and CC51) or animals (CC9, CC20, CC97, CC126, CC133 and CC705) [11,24-27]. Regarding mastitis, bovine strains are usually associated with a few CC (including CC97, CC126, CC130, CC133 and CC705) whose specificity for the mammary gland was either low or undefined [22].

The aim of the present study was to use MLVA for the first time to infer a population structure of *S. aureus* strains from mastitis in dairy cows, goats and sheep from different countries and regions. Through the additional information provided by the analysis of VNTR allele distribution, the objective was also to better identify the evolution and emergence of host-adapted or udder-adapted clones.

## Materials and methods

### Bacterial strains

The 152 strains investigated in this retrospective study were obtained from cases of bovine (*n* = 118), ovine (*n* = 18) and caprine (*n* = 16) clinical or subclinical mastitis. An additional table file shows this in more detail (see Additional file 1). Forty-eight among the bovine strains were collected all over Germany between 2006 and 2009. Nineteen were collected in southern Brazil in 1992 and 1993, and 51 in western France in 2008 and 2009. Sixteen German strains collected in 2009 from different locations were previously described as MRSA ST398 [28]. Characteristics of the 19 Brazilian strains were described by Lange et al. [5]. The ovine and caprine strains were collected from clinical or subclinical mastitis between 1978 and 2010 in France in the main dairy production areas: 16 strains from center-west or south-east for goats and 18 strains from the Pyrenees, the Massif Central or Corsica for ewes. The strains were selected as pure cultures obtained after mastitic milk cultivation on agar plates. Only one strain per herd or flock was considered for this study (see Additional file 1).

### DNA extraction

Strains were cultured overnight at 37 °C in Luria Bertani broth. Genomic DNA was extracted by phenol-chloroform extraction or by using the DNeasy tissue kit (Qiagen, Courtaboeuf, France) with lysostaphin (100 mg/L, Ambi products LLC, USA). Nucleic acid quality and concentration were analysed using an ND-1000 spectrophotometer (NanoDrop, Labtech, Palaiseau, France). Diluted samples of 5 ng/μL in distilled water (Braun, Melsungen, Germany) were used as DNA template for PCR amplification.

### Genotyping data production and analysis

The 16 VNTR loci included in MLVA16_Orsay_ were amplified in two multiplex PCR using the CeeramTools® *Staphylococcus* typing kit (Ceeram, La Chapelle sur Erdre, France) as previously described [22]. The typing data file was imported into BioNumerics version 6.6 (Applied-Maths, Sint-Martens-Latem, Belgium). A cut-off value of 45% similarity was applied to define clusters according to [19]. Simpson’s diversity index was used [29]. The MLVA16_Orsay_ data derived from the 152 strains of this study were compared to published data obtained with the same method [22] in order to tentatively assign the new strains to MLST CC [19,22,30]. In a previous study, 251 *S. aureus* strains isolated from human (*n* = 106), swine (*n* = 32), poultry (*n* = 30), companion animals (*n* = 17), horse (*n* = 5), small ruminant (*n* = 11), rodent (*n* = 2), cattle (*n* = 1), food (*n* = 34) and food poisoning events (*n* = 13) were characterized by MLVA16_Orsay_ complemented by MLST and *spa* typing. This reference dataset is now used to link new strains. All unclustered strains from the present investigation were characterized by MLST and *spa* typing.

The primers and condition used for the *spa* tandem repeat amplification and MLST analysis were as previously described [10,31,32]. The amplicons were purified using the QIAquick PCR purification kit (Qiagen, Courtabœuf, France) and sequenced (Eurofins MWG Operon, Ebersberg, Germany or Beckman-Coulter Genomics, Hertfordshire, UK). The *spa* repeat nomenclature was that of Shopsin et al. [12] and *spa* types were retrieved from [33]. MLST alleles and sequence types (ST) were identified using the MLST database [34].

Data were analysed by chi-square or Student’s *t*-tests. Differences were considered significant when *p* < 0.05.

## Results

### MLVA genotypes and epidemiology

**Figure 1:**
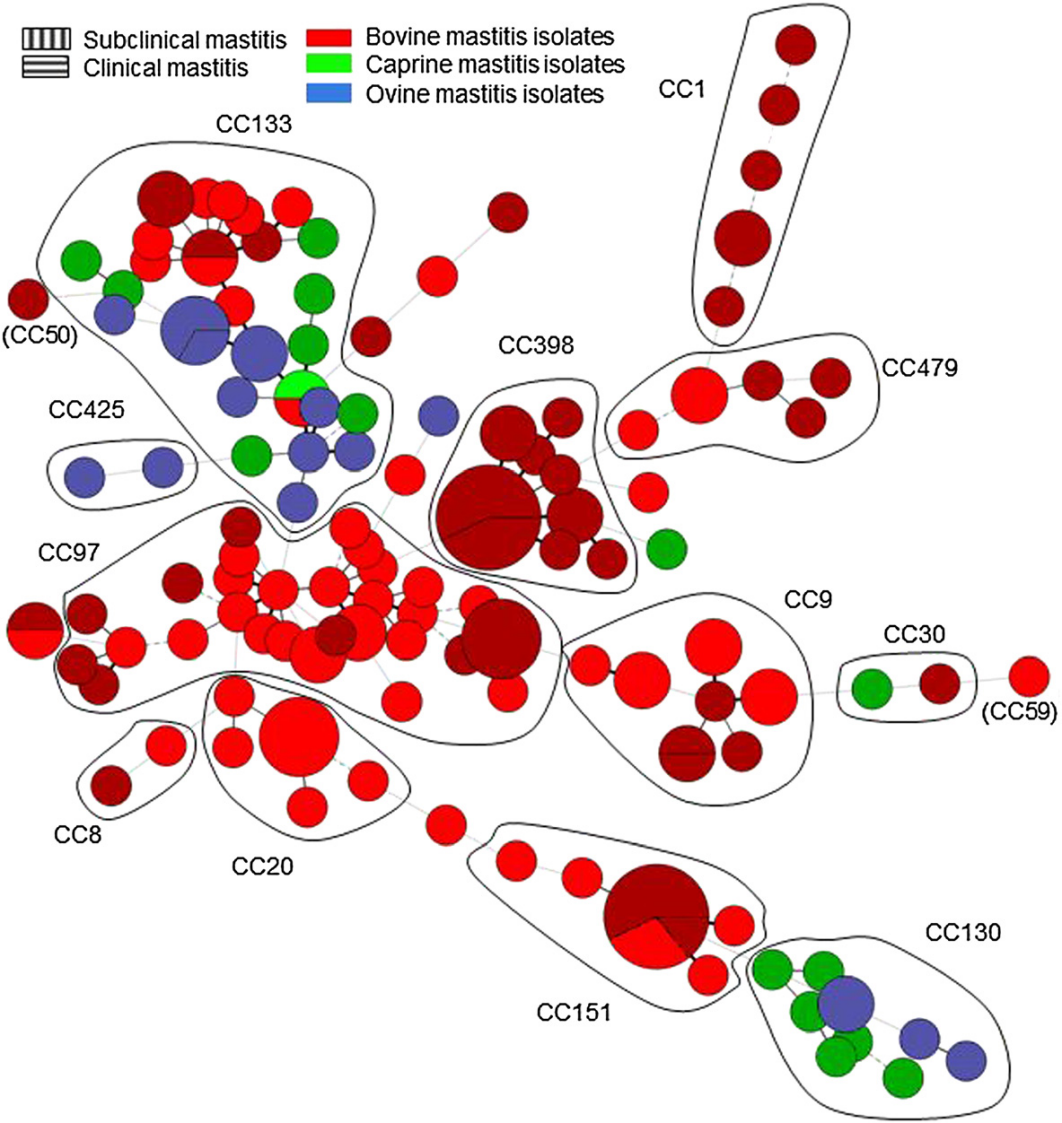
**Minimum spanning tree of the 152** ***S. aureus*** **strains using MLVA16**_**Orsay**_ **.** Each circle represents an MLVA genotype. The genotypes are coloured according to their host. Major CC are indicated.

**Figure 2:**
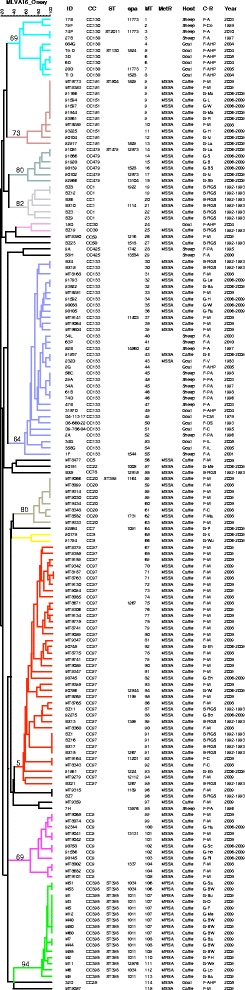
**Dendrogram deduced from the clustering of the 152** ***S. aureus*** **mastitis-associated strains using MLVA16**_**Orsay**_ **.** The colour code reflects MLVA clusters when using the 45% cut off. ID: strain identification. spa: *spa* type. MT: MLVA types. MetR: methicillin-resistance. MRSA: methicillin-resistant *Staphylococcus aureus*. MSSA: methicillin-susceptible *Staphylococcus aureus*. C-R: Country-Region. F: France, A: Aveyron, Co: Corse, PA: Pyrénées-Atlantiques, AHP: Alpes de Haute-Provence, V: Vienne, CR: Charentes-Maritimes, DS: Deux-Sèvres, IL: Indre-et-Loire, M: Manche, C: Calvados, Ma: Mayenne. B: Brazil, RGS: Rio Grande do Sul. G: Germany, Mu: Mutzenich, Lichtenfels: L, Waldeck: W, Marksuhl: Mark. Reinhardshagen: R, Haag: H, Neustadt: N, Langen: La, Sulza: S, Burstadt: B. Bad Soden-Salmunster: BS, Silberfeld: Sb, Lehrte: Le, Babenhausen: Ba, Huttenberg: H, Wiesenthal: Wi, Raesfeld: Ra, Eurasburg: E, Meinhard: Me, Pfronten: P, Kirchhain: K, Wunstorf: Wu, Ehrenberg: EH, Willingen: Wi, Bodelwitz: Bo, Ebersberg: Eb, Hademar: Ha, Schlitz: Sc, Hofbieber: Ho, Ringgau: Ri, Satteldorf: Sa, Baden-Wurttenberg: BW, Bunde: Bu, Osterberg: O, Farven: F, Melle: Me, Bayern: Ba, Petershagen: PH, Westerstede: We, Lorup: Lo.

The 152 ruminant strains were resolved into 115 MLVA genotypes with an overall diversity index of 0.9936. The 118 bovine, 18 ovine and 16 caprine strains belonged to 86, 14 and 15 MLVA genotypes, respectively. One hundred and forty strains fell into twelve clusters, nine of which comprising more than three strains (Figure 1). The clusters were assigned to known MLST-defined CC by comparison with previous MLVA data, *spa* typing and MLST analysis of selected strains. A dendrogram deduced from the clustering of the 152 *S. aureus* mastitis-associated strains is presented in Figure 2. CC97 and CC133 accounted for 22% (34 strains) and 21% (32 strains) of the studied collection, respectively. CC1, CC9, CC20, CC130, CC151, CC398 and CC479 together represented another 45% of the strains. The 16 ST398 MRSA strains were distributed into eight MLVA genotypes and three *spa* types. CC8 and CC30, frequently associated with human *S. aureus* infections, and CC425 a common ovine genotype (Figure 1), were represented by two strains each.

**Figure 3:**
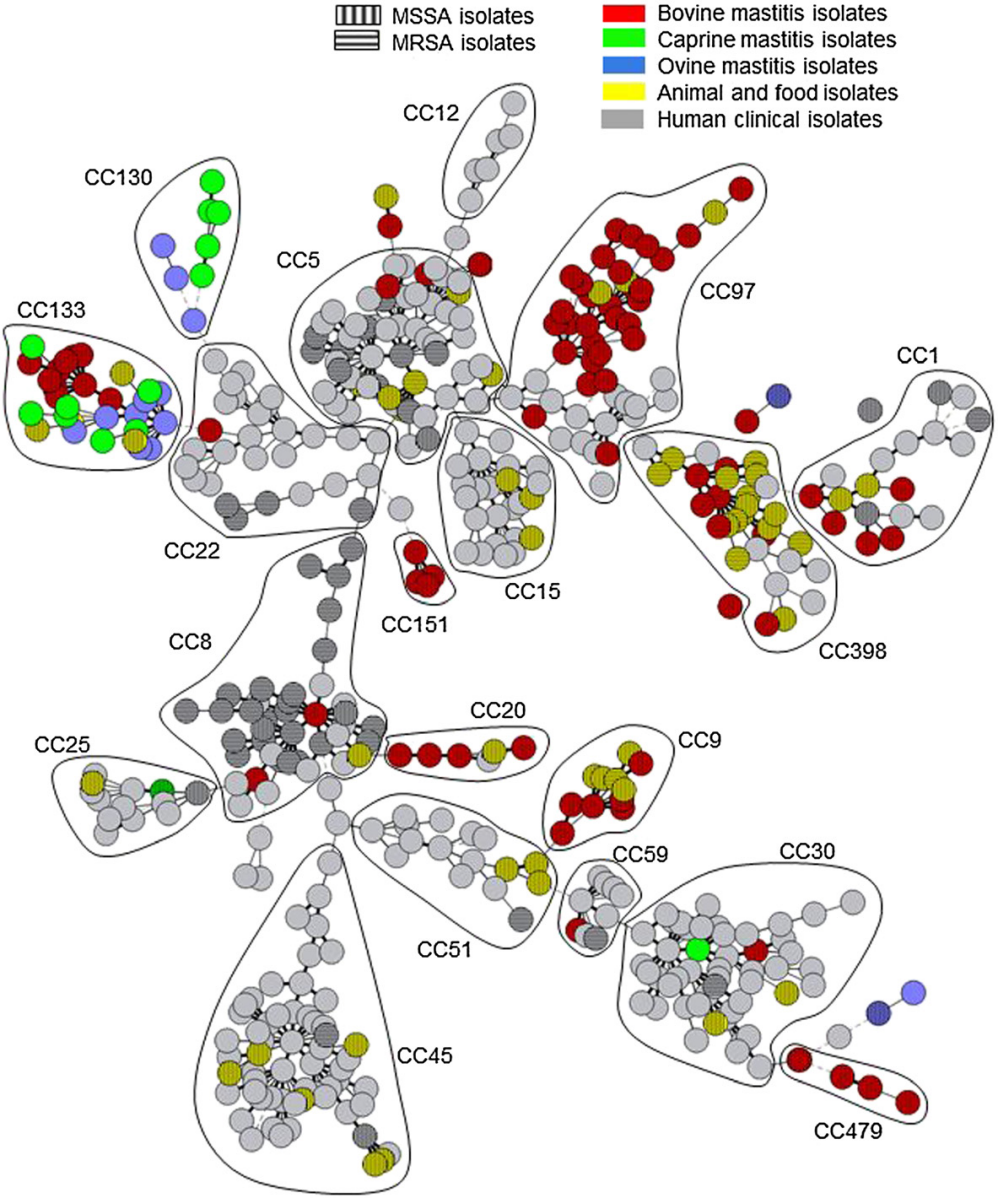
**Mastitis strains superimposed on a background of previously published data from human, animal or food isolates [** 22 **].** Main CC are indicated. MRSA: methicillin-resistant *Staphylococcus aureus*. MSSA: methicillin-susceptible *Staphylococcus aureus*. MRSA and MSSA isolates are highlighted with two different hatch patterns.

Among the singletons, seven could be assigned to CC5, CC7, CC22, CC25, CC50, CC59 or CC78 by comparison with previously typed isolates (Figure 3). Five singletons remained unclustered.

Figure 1 shows the distribution of the strains according to the host. CC130 comprised exclusively small ruminant *S. aureus* strains. Sheep strains (collected from four flocks between 1997 and 2010) and goat strains (collected from six herds in 2004) were segregated by MLVA typing. These ten strains came from three different French husbandry regions without any contact with each other. CC1, CC8, CC9, CC20, CC97, CC151, CC398 and CC479 included only bovine strains. CC133 was the sole cluster showing complete host diversity: 13 bovine strains originating from different collection sites (11% of the bovine isolates), eleven sheep strains (61% of the ovine isolates) and eight goat strains (50% of the caprine strains) belonged to this CC. The remaining small ruminant strains were clustered in CC30, CC425 or were singletons. Only one MLVA genotype comprised strains of two different host species: the cow and goat (MLVA genotype 43, MLST CC133). No MLVA genotype was common to goat and sheep.

Bovine isolates from the three countries fell into 6 up to 10 different CC; small ruminant isolates fell into 3 or 4 CC. The CC distribution was different between Germany, France and Brazil (*p* < 0.001).

Figure 3 shows the comparison of the population structure of *S. aureus* strains from mastitis with the more global population of *S. aureus* strains for which MLVA data was available [22]. Some clusters such as CC8, CC22 and CC45 were highly human specific. CC30 is in an intermediate situation, in agreement with some previous observations [35]. In contrast, CC133 was mainly represented by animal strains, and CC130 by small ruminant mastitis strains. CC97 comprised the largest number of bovine mastitis strains.

### VNTR allele distribution analysis

We calculated the allelic diversity of each VNTR within three groups: human, animal and mammary gland-related strains. The number of alleles per locus was not significantly different between human-related and animal-related clusters, but was significantly reduced in the population of the mammary gland-predominant clusters (*p* < 0.005). The diversity indexes were lower in the latter than in the human-related (*p* < 0.05) or animal-specific (NS) clusters. An additional table file shows this in more detail (see Additional file 2). We also measured the mean number of repetitions per locus and per group. No significant difference was observed between human-predominant and animal-predominant clusters. Differences were noticed when focusing on mammary gland-adapted clusters. This population had a smaller number of repeat units for two VNTR, Sa0122 (*p* < 0.001) and Sa0387 (*p* < 0.0001). An additional file shows this in more detail (see Additional file 3).

## Discussion

### CC130, CC151 and CC479 are strongly associated to mastitis

In the present collection of strains, only 13 (8.6%) from six different lineages (six CC1, two CC8, two CC30 and single CC5, CC22 and CC25) might be of human origin. Among these lineages, CC30 has also been shown to be established in swine [35]. Devriese observed specific phenotypic characteristics associated with animal *S. aureus* strains and subsequently emphasized the existence of ecovars adapted to particular host species [36]. Several studies have identified the existence of *S. aureus* CC that are associated with cows, sheep and goats, and rarely isolated from humans, suggesting that ruminants are the preferred hosts [6,7,9,37]. Conversely, a number of CC shows limited host specificity. Representatives of some animal-predominant CC have spread recently with apparently neither strong host nor geographical barriers. In this study, we reported 35 strains belonging to these well-known CC (16 strains from CC398, eleven strains from CC9 and eight strains from CC20). ST398, a cause of human MRSA infections most often associated with livestock exposure, is hypothesized to have emerged from swine but presumably originates from humans. The jump from humans to livestock was probably accompanied by the acquisition of methicillin and tetracycline resistances [38]. CC20 strains represent approximately 1% of human carriage [39,40] and infections [41] but are also often sampled from cow mastitic milk [26,42,43].

Almost half of the collection studied belonged to two major animal-predominant CC (CC97 and CC133). Twenty-nine and 26 MLVA16 genotypes were observed for a total of 34 CC97 strains and 32 CC133 strains respectively. CC97 is a widespread bovine lineage largely responsible for bovine mastitis cases in Chile, Brazil, Japan and the United States [13,44], and also recently isolated from human [45] and porcine hosts [46]. CC133 is commonly sampled from milk produced by small ruminants [6,47] and by cows (15 among the 33 CC133 strains are from bovines) [37,48] suffering from mastitis.

In the present study, we identified three additional clusters putatively showing strong association with the mammary gland tissue: the “bovine” CC151 (eleven strains) and CC479 (six strains), and the “small ruminants” CC130 (ten strains). All together, they represent 18% of the present collection. These lineages have almost never been isolated from humans and, moreover, were almost exclusively sampled from intramammary infections [25,37,49-51], with one published exception (nasal cavity) [52]. The rare CC479 was not mentioned as mastitis-associated [22]. Interestingly, whole genome analysis demonstrated that CC130 and CC151 are closely related [49] in agreement with MLVA clustering. Several studies revealed that a few CC are responsible for most mastitis cases [6,7,9]. Van Leeuwen et al. hypothesized that traits shared by bovine and small ruminant mastitis strains were related to tissue specificity. This would explain that mastitis-associated strains from these farm animal species formed a distinct genetic cluster [53]. These CC have a broad geographical distribution: in the present study, the CC151-MLVA genotype 2-5-3-1-2-2-2-1-9-3-7-0.5-1-0.5-3-1 was shown to be shared by strains sampled in Germany and France.

From the population structure point of view, one must remain careful when interpreting the observation of a small number of CC comprising the majority of livestock and especially mammary isolates. Recently, a single publication identified 9 CC and 4 ST in ovine and caprine mastitis [51], whereas the variability of small ruminant mammary CC was previously thought to be restricted to two CC (CC130, CC133) according to a review published earlier in the same year [27]. Thus, for each host species, the size of the world target population (livestock) and of the analyzed samples is important to consider, just like its representativeness and the variability of the husbandry systems. For instance the literature on small ruminant *S. aureus* carriage is very limited as compared to humans.

### VNTR as genetic markers to infer lineage emergence

It has been proposed that a low level of VNTR genetic diversity inside a lineage may reflect recent emergence, and that there exists a tendency toward the shortening of tandem repeat array during evolution (such as in *Mycobacterium tuberculosis*, [54]). Interestingly, we noticed that the mammary gland-predominant lineages (CC130, C151 and CC479) exhibit a smaller repeat unit number per locus and a lower diversity index as compared to other lineages. These observations are most striking in the case of CC151 (Additional file 3). Modifications in industrial livestock husbandry of dairy ruminants could have led to modified access of microbial flora to mammary glands leading to the emergence of some CC such as CC151. Analysis of RF122 strain (ST151) genome sequence provided evidence that this mammary-gland-adapted strain had recently diversified from an ancestor with a supposed human origin through acquisition of mobile genetic elements and gene decay [55,56]. Because tandem repeat mutation rates have been suggested to vary within different lineages in some bacterial species [57], whole genome sequence analysis of well-chosen strains will be necessary to correlate tandem repeat diversity and more neutral genome diversity in *S. aureus*. Indeed in a preliminary study, we observed that at least some tandem repeats may have an effect on the transcription level of adjacent genes indicating that they are not neutral. Within the same CC, the transcription level of the gene located immediately downstream from Sa0906 was five-fold higher in a strain with four repeat units as compared to a strain with one repeat unit (Vergnaud et al. unpublished; [58]).

### Interest of MLVA as a subtyping tool for improving the efficiency of mastitis control

The characterization of mastitis transmission models (contagious *versus* environmental herd mastitis) is a critical point to implement relevant control measures. In the case of *S. aureus*, generally classified as a contagious pathogen, the molecular epidemiology profile is in some herds of the environmental type [59]. Taking advantage of the possibility offered by MLVA to efficiently discriminate field isolates, we suggest comparing, in seriously affected herds, various isolates originating from intramammary infections, teat skin, milking machine clusters and the environment. Fournier et al. [60] demonstrated an association between genotypes and mastitis clinical outcome. This original result regarding the accessory genome (presence or absence of toxins) would benefit from the application of the core-genome genotyping achieved by MLVA for identification of particular epidemic or virulent strains. The relatively low cost of MLVA typing and the availability of freely accessible databases on the internet may help enlarge our knowledge on these points [61,62].

## Additional files

Additional file 1:
**List of** ***Staphylococcus aureus*** **strains used in the study and genotyping data.** For each strain, host, isolation year, geographic origin (region and country), mastitis clinical type, collection origin, CC and MLVA type are given. The 152 strains were isolated between 1992 and 2009 in three countries (Germany, France and Brazil) from mastitic-milk of cows, ewes and goats. Additional file 2:
**Allelic richness and diversity per locus for each group of strains (human-related, animal-specific, mammary gland-adapted).** For the three groups of strains, the number of alleles and the diversity index are given for each VNTR. Additional file 3:
**Mean number of repeat units per locus and for each group (human-predominant, animal-predominant, mammary gland-predominant).** For each VNTR, the number of repeat units (mean and standard deviation) are presented for human-, animal- and mammary gland (mastitis)-predominant strains. CC151, belonging to the latter group, is also presented alone.
