# Supplementary material for: Staphylococcus aureus from 152 cases of bovine, ovine and caprine mastitis investigated by Multiple-locus variable number of tandem repeat analysis (MLVA)
Source: Vet Res. 2014 Oct 2;45(1):97. doi: 10.1186/s13567-014-0097-4 (PMC4195859; doi:10.1186/s13567-014-0097-4)
Supplement: Additional file 1: — List of Staphylococcus aureus strains used in the study and genotyping data. For each strain, host, isolation year, geographic origin (region and country), mastitis clinical type, collection origin, CC and MLVA type are given. The 152 strains were isolated between 1992 and 2009 in three countries (Germany, France and Brazil) from mastitic-milk of cows, ewes and goats. [file 13567_2014_97_MOESM1_ESM.doc]

| **Strain ID** | **Host** | **Isolation year** | **Geographic origin** | **Mastitis clinical type** | **Collection origin** | **CC** | **MLVA type** |
| --- | --- | --- | --- | --- | --- | --- | --- |
| BZ8 | Cattle | 1992-1993 | Rio Grande do Sul, Brazil | Subclinical mastitis | FLI, S. SCHWARZ | CC1 | 19 |
| BZ12 | Cattle | 1992-1993 | Rio Grande do Sul, Brazil | Subclinical mastitis | FLI, S. SCHWARZ | CC1 | 19 |
| BZ6 | Cattle | 1992-1993 | Rio Grande do Sul, Brazil | Subclinical mastitis | FLI, S. SCHWARZ | CC1 | 20 |
| BZ10 | Cattle | 1992-1993 | Rio Grande do Sul, Brazil | Subclinical mastitis | FLI, S. SCHWARZ | CC1 | 21 |
| BZ3 | Cattle | 1992-1993 | Rio Grande do Sul, Brazil | Subclinical mastitis | FLI, S. SCHWARZ | CC1 | 22 |
| BZ9 | Cattle | 1992-1993 | Rio Grande do Sul, Brazil | Subclinical mastitis | FLI, S. SCHWARZ | CC1 | 23 |
| BZ19 | Cattle | 1992-1993 | Rio Grande do Sul, Brazil | Subclinical mastitis | FLI, S. SCHWARZ | CC30 | 25 |
| BZ23 | Cattle | 1992-1993 | Rio Grande do Sul, Brazil | Subclinical mastitis | FLI, S. SCHWARZ | CC50 | 27 |
| BZ4 | Cattle | 1992-1993 | Rio Grande do Sul, Brazil | Subclinical mastitis | FLI, S. SCHWARZ | CC133 | 30 |
| BZ15 | Cattle | 1992-1993 | Rio Grande do Sul, Brazil | Subclinical mastitis | FLI, S. SCHWARZ | CC133 | 30 |
| BZ5 | Cattle | 1992-1993 | Rio Grande do Sul, Brazil | Subclinical mastitis | FLI, S. SCHWARZ | CC78 | 58 |
| BZ11 | Cattle | 1992-1993 | Rio Grande do Sul, Brazil | Subclinical mastitis | FLI, S. SCHWARZ | CC97 | 87 |
| BZ13 | Cattle | 1992-1993 | Rio Grande do Sul, Brazil | Subclinical mastitis | FLI, S. SCHWARZ | CC97 | 89 |
| BZ1 | Cattle | 1992-1993 | Rio Grande do Sul, Brazil | Subclinical mastitis | FLI, S. SCHWARZ | CC97 | 91 |
| BZ16 | Cattle | 1992-1993 | Rio Grande do Sul, Brazil | Subclinical mastitis | FLI, S. SCHWARZ | CC97 | 91 |
| BZ17 | Cattle | 1992-1993 | Rio Grande do Sul, Brazil | Subclinical mastitis | FLI, S. SCHWARZ | CC97 | 91 |
| BZ18 | Cattle | 1992-1993 | Rio Grande do Sul, Brazil | Subclinical mastitis | FLI, S. SCHWARZ | CC97 | 91 |
| BZ21 | Cattle | 1992-1993 | Rio Grande do Sul, Brazil | Subclinical mastitis | FLI, S. SCHWARZ | CC97 | 95 |
| BZ7 | Cattle | 1992-1993 | Rio Grande do Sul, Brazil | Subclinical mastitis | FLI, S. SCHWARZ |  | 96 |
| 91844 | Cattle | 2006-2009 | Marksuhl, Germany | Subclinical mastitis | FLI, S. SCHWARZ | CC151 | 9 |
| 91920 | Cattle | 2006-2009 | Bürstadt, Germany | Subclinical mastitis | FLI, S. SCHWARZ | CC479 | 15 |
| 90139 | Cattle | 2006-2009 | Bad Soden-Salmünster, Germany | Subclinical mastitis | FLI, S. SCHWARZ | CC479 | 16 |
| 92966 | Cattle | 2006-2009 | Silberfeld, Germany | Subclinical mastitis | FLI, S. SCHWARZ | CC479 | 18 |
| 90745 | Cattle | 2006-2009 | Ehrenberg, Germany | Subclinical mastitis | FLI, S. SCHWARZ | CC97 | 82 |
| 90756 | Cattle | 2006-2009 | Willingen, Germany | Subclinical mastitis | FLI, S. SCHWARZ | CC97 | 84 |
| 92275 | Cattle | 2006-2009 | Bodelwitz, Germany | Subclinical mastitis | FLI, S. SCHWARZ | CC97 | 88 |
| 91591 | Cattle | 2006-2009 | Ebersberg, Germany | Subclinical mastitis | FLI, S. SCHWARZ | CC97 | 93 |
| 92344 | Cattle | 2006-2009 | Hademar, Germany | Subclinical mastitis | FLI, S. SCHWARZ | CC9 | 100 |
| 90758 | Cattle | 2006-2009 | Schlitz, Germany | Subclinical mastitis | FLI, S. SCHWARZ | CC9 | 102 |
| M51 | Cattle | 2009 | Satteldorf, Germany | Subclinical mastitis | FLI, S. SCHWARZ | CC398 | 106 |
| M53 | Cattle | 2009 | Baden-Württemberg, Germany | Subclinical mastitis | FLI, S. SCHWARZ | CC398 | 106 |
| M40 | Cattle | 2009 | Baden-Württemberg, Germany | Subclinical mastitis | FLI, S. SCHWARZ | CC398 | 107 |
| M50 | Cattle | 2009 | Baden-Württemberg, Germany | Subclinical mastitis | FLI, S. SCHWARZ | CC398 | 107 |
| M60 | Cattle | 2009 | Baden-Württemberg, Germany | Subclinical mastitis | FLI, S. SCHWARZ | CC398 | 107 |
| M7 | Cattle | 2009 | Bayern, Germany | Subclinical mastitis | FLI, S. SCHWARZ | CC398 | 108 |
| M44 | Cattle | 2009 | Baden-Württemberg, Germany | Subclinical mastitis | FLI, S. SCHWARZ | CC398 | 108 |
| M58 | Cattle | 2009 | Baden-Württemberg, Germany | Subclinical mastitis | FLI, S. SCHWARZ | CC398 | 109 |
| M9 | Cattle | 2009 | Bayern, Germany | Subclinical mastitis | FLI, S. SCHWARZ | CC398 | 113 |
| 93225 | Cattle | 2006-2009 | Haag, Germany | Mastitis | FLI, S. SCHWARZ | CC151 | 11 |
| 90104 | Cattle | 2006-2009 | Neustadt, Germany | Mastitis | FLI, S. SCHWARZ | CC151 | 12 |
| 92917 | Cattle | 2006-2009 | Langen, Germany | Mastitis | FLI, S. SCHWARZ | CC151 | 13 |
| 91051 | Cattle | 2006-2009 | Langen, Germany | Mastitis | FLI, S. SCHWARZ | CC479 | 14 |
| 91866 | Cattle | 2006-2009 | Sulza, Germany | Mastitis | FLI, S. SCHWARZ | CC479 | 14 |
| 90102 | Cattle | 2006-2009 | Neustadt, Germany | Mastitis | FLI, S. SCHWARZ | CC479 | 17 |
| 91793 | Cattle | 2006-2009 | Lehrte, Germany | Mastitis | FLI, S. SCHWARZ | CC133 | 32 |
| 90088 | Cattle | 2006-2009 | Wiesenthal, Germany | Mastitis | FLI, S. SCHWARZ | CC133 | 35 |
| 90105 | Cattle | 2006-2009 | Raesfeld, Germany | Mastitis | FLI, S. SCHWARZ | CC133 | 36 |
| 91537 | Cattle | 2006-2009 | Eurasburg, Germany | Mastitis | FLI, S. SCHWARZ | CC133 | 43 |
| 92554 | Cattle | 2006-2009 | Pfronten, Germany | Mastitis | FLI, S. SCHWARZ | CC7 | 64 |
| 91794 | Cattle | 2006-2009 | Wunstorf, Germany | Mastitis | FLI, S. SCHWARZ | CC8 | 66 |
| 91166 | Cattle | 2006-2009 | Mützenich, Germany | Clinical mastitis | FLI, S. SCHWARZ | CC151 | 9 |
| 91594 | Cattle | 2006-2009 | Lichtenfels, Germany | Clinical mastitis | FLI, S. SCHWARZ | CC151 | 9 |
| 91597 | Cattle | 2006-2009 | Waldeck, Germany | Clinical mastitis | FLI, S. SCHWARZ | CC151 | 9 |
| 93861 | Cattle | 2006-2009 | Reinhardshagen, Germany | Clinical mastitis | FLI, S. SCHWARZ | CC151 | 9 |
| 93822 | Cattle | 2006-2009 | Babenhausen, Germany | Clinical mastitis | FLI, S. SCHWARZ | CC133 | 32 |
| 91592 | Cattle | 2006-2009 | Hüttenberg, Germany | Clinical mastitis | FLI, S. SCHWARZ | CC133 | 34 |
| 90181 | Cattle | 2006-2009 | Meinhard, Germany | Clinical mastitis | FLI, S. SCHWARZ | CC22 | 57 |
| 90178 | Cattle | 2006-2009 | Kirchhain, Germany | Clinical mastitis | FLI, S. SCHWARZ | CC8 | 65 |
| 91586 | Cattle | 2006-2009 | Hofbieber, Germany | Clinical mastitis | FLI, S. SCHWARZ | CC9 | 102 |
| 90145 | Cattle | 2006-2009 | Ringgau, Germany | Clinical mastitis | FLI, S. SCHWARZ | CC9 | 103 |
| M1 | Cattle | 2009 | Bünde, Germany | Clinical mastitis | FLI, S. SCHWARZ | CC398 | 107 |
| M3 | Cattle | 2009 | Osterberg, Germany | Clinical mastitis | FLI, S. SCHWARZ | CC398 | 107 |
| M5 | Cattle | 2009 | Farven, Germany | Clinical mastitis | FLI, S. SCHWARZ | CC398 | 107 |
| M12 | Cattle | 2009 | Melle, Germany | Clinical mastitis | FLI, S. SCHWARZ | CC398 | 107 |
| M2 | Cattle | 2009 | Petershagen, Germany | Clinical mastitis | FLI, S. SCHWARZ | CC398 | 110 |
| M11 | Cattle | 2009 | Westerstede, Germany | Clinical mastitis | FLI, S. SCHWARZ | CC398 | 111 |
| M6 | Cattle | 2009 | Lorup, Germany | Clinical mastitis | FLI, S. SCHWARZ | CC398 | 112 |
| MT8773 | Cattle | 2008 | Montmartin-en-Graignes, Manche, France | Mastitis | LDA50, M. TREILLES | CC151 | 9 |
| MT8259 | Cattle | 2008 | Saint-Pierre-Langers, Manche, France | Mastitis | LDA50, M. TREILLES | CC151 | 10 |
| MT8168 | Cattle | 2008 | Acqueville, Manche, France | Mastitis | LDA50, M. TREILLES | CC133 | 31 |
| MT8251 | Cattle | 2008 | Saint-Romphaire, Manche, France | Mastitis | LDA50, M. TREILLES | CC133 | 33 |
| MT8141 | Cattle | 2008 | Lessay, Manche, France | Mastitis | LDA50, M. TREILLES | CC133 | 37 |
| MT8064 | Cattle | 2008 | Auvers, Manche, France | Mastitis | LDA50, M. TREILLES | CC133 | 38 |
| MT8604 | Cattle | 2008 | Camprond, Manche, France | Mastitis | LDA50, M. TREILLES | CC133 | 39 |
| MT8477 | Cattle | 2008 | Courcy, Manche, France | Mastitis | LDA50, M. TREILLES | CC5 | 56 |
| MT8066 | Cattle | 2008 | Saint-Laurent-de-Terregatte, Manche, France | Mastitis | LDA50, M. TREILLES | CC20 | 59 |
| MT8099 | Cattle | 2008 | Saint-Pair-sur-Mer, Manche, France | Mastitis | LDA50, M. TREILLES | CC20 | 59 |
| MT8314 | Cattle | 2008 | Sartilly, Manche, France | Mastitis | LDA50, M. TREILLES | CC20 | 59 |
| MT8434 | Cattle | 2008 | Carquebut, Manche, France | Mastitis | LDA50, M. TREILLES | CC20 | 60 |
| MT8348 | Cattle | 2008 | Quetteville, Calvados, France | Mastitis | LDA50, M. TREILLES | CC20 | 61 |
| MT8582 | Cattle | 2008 | Fougerolles-du-Plessis, Mayenne, France | Mastitis | LDA50, M. TREILLES | CC20 | 62 |
| MT8333 | Cattle | 2008 | Saint-Cyr-du-Bailleul, Manche, France | Mastitis | LDA50, M. TREILLES | CC20 | 63 |
| MT8189 | Cattle | 2008 | Muneville-sur-Mer, Manche, France | Mastitis | LDA50, M. TREILLES | CC97 | 69 |
| MT8187 | Cattle | 2008 | Muneville-sur-Mer, Manche, France | Mastitis | LDA50, M. TREILLES | CC97 | 71 |
| MT8763 | Cattle | 2008 | Parigny, Manche, France | Mastitis | LDA50, M. TREILLES | CC97 | 71 |
| MT8130 | Cattle | 2008 | Pont-Hébert, Manche, France | Mastitis | LDA50, M. TREILLES | CC97 | 72 |
| MT8085 | Cattle | 2008 | Hauteville-la-Guichard, Manche, France | Mastitis | LDA50, M. TREILLES | CC97 | 74 |
| MT8671 | Cattle | 2008 | Saint-Jean-des-Champs, Manche, France | Mastitis | LDA50, M. TREILLES | CC97 | 75 |
| MT8134 | Cattle | 2008 | Cambernon, Manche, France | Mastitis | LDA50, M. TREILLES | CC97 | 77 |
| MT8775 | Cattle | 2008 | Saint-Lô, Manche, France | Mastitis | LDA50, M. TREILLES | CC97 | 78 |
| MT8741 | Cattle | 2008 | Saint-Georges-de-Bohon, Manche, France | Mastitis | LDA50, M. TREILLES | CC97 | 79 |
| MT8059 | Cattle | 2008 | Saint-Sauveur, Manche, France | Mastitis | LDA50, M. TREILLES | CC97 | 80 |
| MT8395 | Cattle | 2008 | Le Mesnil-Villeman, Manche, France | Mastitis | LDA50, M. TREILLES | CC97 | 83 |
| MT8269 | Cattle | 2008 | Baudreville, Manche, France | Mastitis | LDA50, M. TREILLES | CC97 | 85 |
| MT8765 | Cattle | 2008 | Saint-Jean-des-Champs, Manche, France | Mastitis | LDA50, M. TREILLES | CC97 | 86 |
| MT8360 | Cattle | 2008 | Montgothier, Manche, France | Mastitis | LDA50, M. TREILLES | CC97 | 90 |
| MT8164 | Cattle | 2008 | Drubec, Calvados, France | Mastitis | LDA50, M. TREILLES | CC97 | 92 |
| MT8343 | Cattle | 2008 | Drubec, Calvados, France | Mastitis | LDA50, M. TREILLES | CC97 | 92 |
| MT8065 | Cattle | 2008 | Saint-Laurent-de-Terregatte, Manche, France | Mastitis | LDA50, M. TREILLES | CC9 | 99 |
| MT8074 | Cattle | 2008 | Saint-Laurent-de-Terregatte, Manche, France | Mastitis | LDA50, M. TREILLES | CC9 | 99 |
| MT8041 | Cattle | 2008 | Chèvreville, Manche, France | Mastitis | LDA50, M. TREILLES | CC9 | 101 |
| MT8042 | Cattle | 2008 | Chèvreville, Manche, France | Mastitis | LDA50, M. TREILLES | CC9 | 101 |
| MT8002 | Cattle | 2008 | Saint-Jean-de-Daye, Manche, France | Mastitis | LDA50, M. TREILLES | CC9 | 104 |
| MT8682 | Cattle | 2008 | Saint-Côme-du-Mont, Manche, France | Mastitis | LDA50, M. TREILLES | CC9 | 104 |
| MT8101 | Cattle | 2008 | Saint-Côme-du-Mont, Manche, France | Mastitis | LDA50, M. TREILLES | CC9 | 105 |
| MT9353 | Cattle | 2009 | Remilly-sur-Lozon, Manche, France | Mastitis | LDA50, M. TREILLES | CC151 | 9 |
| MT9350 | Cattle | 2009 | Remilly-sur-Lozon, Manche, France | Mastitis | LDA50, M. TREILLES | CC59 | 26 |
| MT9030 | Cattle | 2009 | Coudeville-sur-Mer, Manche, France | Mastitis | LDA50, M. TREILLES | CC20 | 59 |
| MT9379 | Cattle | 2009 | Saint-Brice-de-Landelles, Manche, France | Mastitis | LDA50, M. TREILLES | CC97 | 67 |
| MT9385 | Cattle | 2009 | Feugères, Manche, France | Mastitis | LDA50, M. TREILLES | CC97 | 68 |
| MT9342 | Cattle | 2009 | Rocheville, Manche, France | Mastitis | LDA50, M. TREILLES | CC97 | 70 |
| MT9094 | Cattle | 2009 | Saint-Martin-le-Bouillant, Manche, France | Mastitis | LDA50, M. TREILLES | CC97 | 73 |
| MT9306 | Cattle | 2009 | Le Teilleul, Manche, France | Mastitis | LDA50, M. TREILLES | CC97 | 76 |
| MT9347 | Cattle | 2009 | Bacilly, Manche, France | Mastitis | LDA50, M. TREILLES | CC97 | 81 |
| MT9279 | Cattle | 2009 | Subligny, Manche, France | Mastitis | LDA50, M. TREILLES | CC97 | 94 |
| MT9315 | Cattle | 2009 | Saint-Michel-de-Montjoie, Manche, France | Mastitis | LDA50, M. TREILLES |  | 96 |
| MT9359 | Cattle | 2009 | Reffuveille, Manche, France | Mastitis | LDA50, M. TREILLES |  | 97 |
| MT9057 | Cattle | 2009 | Herqueville, Manche, France | Mastitis | LDA50, M. TREILLES |  | 115 |
| 232D | Goat | 1983 | Vienne, France | Mastitis | ANSES, M.-L. DE BUYSER | CC133 | 43 |
| D4-113-17 | Goat | 1978 | Charente-Maritime, France | Subclinical mastitis | INRA ISP, F. GILBERT | CC133 | 49 |
| D8-660-22 | Goat | 1993 | Deux-Sèvres, France | Subclinical mastitis | INRA ISP, F. GILBERT | CC133 | 50 |
| D9-786-04 | Goat | 1995 | Cher, France | Subclinical mastitis | INRA ISP, F. GILBERT | CC133 | 51 |
| 084G | Goat | 2004 | Alpes de Haute Provence, France | Subclinical mastitis | INRA ISP, F. GILBERT | CC130 | 4 |
| Touffue D | Goat | 2004 | Alpes de Haute Provence, France | Subclinical mastitis | INRA ISP, F. GILBERT | CC130 | 5 |
| 6D | Goat | 2004 | Alpes de Haute Provence, France | Subclinical mastitis | INRA ISP, F. GILBERT | CC130 | 6 |
| Timili D | Goat | 2004 | Alpes de Haute Provence, France | Subclinical mastitis | INRA ISP, F. GILBERT | CC130 | 8 |
| 15D | Goat | 2004 | Alpes de Haute Provence, France | Subclinical mastitis | INRA ISP, F. GILBERT | CC30 | 24 |
| 3187D | Goat | 2004 | Alpes de Haute Provence, France | Subclinical mastitis | INRA ISP, F. GILBERT | CC133 | 48 |
| 3D | Goat | 2005 | Alpes de Haute Provence, France | Subclinical mastitis | INRA ISP, F. GILBERT | CC130 | 6 |
| 20D | Goat | 2005 | Alpes de Haute Provence, France | Subclinical mastitis | INRA ISP, F. GILBERT | CC130 | 7 |
| 2G | Goat | 2005 | Alpes de Haute Provence, France | Subclinical mastitis | INRA ISP, F. GILBERT | CC133 | 44 |
| 32D | Goat | 2005 | Alpes de Haute Provence, France | Subclinical mastitis | INRA ISP, F. GILBERT | CC25 | 114 |
| 34G | Goat | 2005 | Sainte-Maure, Indre et Loire, France | Subclinical mastitis | INRA ISP, F. GILBERT | CC133 | 53 |
| 358G | Goat | 2005 | Sainte-Maure, Indre et Loire, France | Subclinical mastitis | INRA ISP, F. GILBERT | CC133 | 54 |
| 27B | Sheep | 1997 | Aveyron, France | Clinical mastitis | INRA-ENVT, D. BERGONIER | CC130 | 3 |
| 52B | Sheep | 1997 | Aveyron, France | Clinical mastitis | INRA-ENVT, D. BERGONIER | CC133 | 42 |
| 47B | Sheep | 2003 | Aveyron, France | Clinical mastitis | INRA-ENVT, D. BERGONIER | CC133 | 47 |
| 74P | Sheep | 2010 | Aveyron, France | Clinical mastitis | INRA-ENVT, D. BERGONIER | CC130 | 3 |
| 63P | Sheep | 2010 | Aveyron, France | Clinical mastitis | INRA-ENVT, D. BERGONIER | CC133 | 41 |
| 58H | Sheep | 2000 | Aveyron, France | Subclinical mastitis | INRA-ENVT, D. BERGONIER | CC425 | 29 |
| 1F | Sheep | 2001 | Aveyron, France | Subclinical mastitis | INRA-ENVT, D. BERGONIER | CC133 | 55 |
| 17B | Sheep | 2003 | Aveyron, France | Subclinical mastitis | INRA-ENVT, D. BERGONIER | CC130 | 1 |
| 34L | Sheep | 2003 | Aveyron, France | Subclinical mastitis | INRA-ENVT, D. BERGONIER | CC133 | 40 |
| 79F | Sheep | 1998 | Corse, France | Clinical mastitis | INRA-ENVT, D. BERGONIER | CC130 | 2 |
| 2A | Sheep | 1996 | Pyrénées-Atlantiques, France | Clinical mastitis | INRA-ENVT, D. BERGONIER | CC133 | 52 |
| 34A | Sheep | 1997 | Pyrénées-Atlantiques, France | Clinical mastitis | INRA-ENVT, D. BERGONIER | CC133 | 45 |
| 29A | Sheep | 2003 | Pyrénées-Atlantiques, France | Clinical mastitis | INRA-ENVT, D. BERGONIER | CC133 | 45 |
| 9A | Sheep | 1995 | Pyrénées-Atlantiques, France | Subclinical mastitis | INRA-ENVT, D. BERGONIER | CC425 | 28 |
| 74D | Sheep | 1996 | Pyrénées-Atlantiques, France | Subclinical mastitis | INRA-ENVT, D. BERGONIER | CC133 | 46 |
| 7H | Sheep | 1996 | Pyrénées-Atlantiques, France | Subclinical mastitis | INRA-ENVT, D. BERGONIER |  | 98 |
| 56C | Sheep | 1998 | Pyrénées-Atlantiques, France | Subclinical mastitis | INRA-ENVT, D. BERGONIER | CC133 | 45 |
| 61B | Sheep | 1998 | Pyrénées-Atlantiques, France | Subclinical mastitis | INRA-ENVT, D. BERGONIER | CC133 | 46 |
