# Supplementary material for: Staphylococcus aureus from 152 cases of bovine, ovine and caprine mastitis investigated by Multiple-locus variable number of tandem repeat analysis (MLVA)
Source: Vet Res. 2014 Oct 2;45(1):97. doi: 10.1186/s13567-014-0097-4 (PMC4195859; doi:10.1186/s13567-014-0097-4)
Supplement: Additional file 2: — Allelic richness and diversity per locus for each group of strains (human-related, animal-specific, mammary gland-adapted). For the three groups of strains, the number of alleles and the diversity index are given for each VNTR. [file 13567_2014_97_MOESM2_ESM.doc]

| **VNTR** | **Human-predominant**  **(Number of alleles -**  **Diversity index** [**standard deviation of 5%**]**)** | **Animal-predominant**  **(Number of alleles -**  **Diversity index** [**standard deviation of 5%**]**)** | **Mammary gland-predominant**  **(Number of alleles -**  **Diversity index** [**standard deviation of 5%**]**)** |
| --- | --- | --- | --- |
| Sa0122 | 12 - 0.7833 [0.7446,0.8219] | 13 - 0.8474 [0.8226,0.8722] | 5 - 0.6182 [0.4758,0.7607] |
| Sa0311 | 5 - 0.6683 [0.6272,0.7093] | 5 - 0.6346 [0.5711,0.6981] | 2 - 0.4843 [0.3880,0.5807] |
| Sa0387 | 7 - 0.7482 [0.7115,0.7849] | 6 - 0.5829 [0.5211,0.6446] | 2 - 0.4615 [0.3406,0.5825] |
| Sa0550 | 4 - 0.3981 [0.3127,0.4836] | 2 - 0.0106 [0.0000,0.0315] | 1 - 0.0000 [0.0000,0.0000] |
| Sa0684 | 4 - 0.6125 [0.5601,0.6648] | 3 - 0.4388 [0.3626,0.5150] | 2 - 0.3590 [0.1812,0.5368] |
| Sa0964 | 6 - 0.7033 [0.6737,0.7329] | 7 - 0.7393 [0.7096,0.7689] | 4 - 0.5641 [0.3873,0.7409] |
| Sa1097 | 15 - 0.7823 [0.7308,0.8338] | 8 - 0.7130 [0.6829,0.7430] | 3 - 0.6724 [0.6173,0.7274] |
| Sa1194 | 7 - 0.6965 [0.6575,0.7354] | 8 - 0.6967 [0.6659,0.7274] | 6 - 0.7721 [0.7001,0.8440] |
| Sa1729 | 7 - 0.7121 [0.6725,0.7517] | 8 - 0.7490 [0.7157,0.7824] | 4 - 0.6980 [0.6283,0.7677] |
| Sa1866 | 4 - 0.4908 [0.4141,0.5675] | 4 - 0.2906 [0.2132,0.3680] | 6 - 0.7436 [0.6439,0.8433] |
| Sa0266 | 6 - 0.6475 [0.6130,0.6820] | 7 - 0.6805 [0.6433,0.7178] | 4 - 0.5726 [0.4606,0.6847] |
| Sa0704 | 9 - 0.8115 [0.7878,0.8351] | 6 - 0.7378 [0.7111,0.7646] | 4 - 0.5185 [0.3347,0.7024] |
| Sa1132 | 10 - 0.7040 [0.6612,0.7467] | 7 - 0.7296 [0.6986,0.7605] | 6 - 0.7835 [0.7020,0.8649] |
| Sa1291 | 9 - 0.6957 [0.6427,0.7487] | 11 - 0.8314 [0.8079,0.8549] | 6 - 0.7607 [0.6759,0.8455] |
| Sa2039 | 6 - 0.6880 [0.6370,0.7390] | 5 - 0.5326 [0.4660,0.5993] | 5 - 0.6610 [0.4947,0.8272] |
| Sa2511 | 14 - 0.8760 [0.8555,0.8966] | 11 - 0.7719 [0.7338,0.8099] | 7 - 0.7692 [0.6651,0.8733] |
| MLVA-16_Orsay_ | 105 - 0.9863 [0.9794,0.9931] | 106 - 0.9755 [0.9651,0.9859] | 18 - 0.9316 [0.8595,1.0000] |
