# Supplementary figures and images for: Staphylococcus aureus from 152 cases of bovine, ovine and caprine mastitis investigated by Multiple-locus variable number of tandem repeat analysis (MLVA)
Source: Vet Res. 2014 Oct 2;45(1):97. doi: 10.1186/s13567-014-0097-4 (PMC4195859; doi:10.1186/s13567-014-0097-4)

## Slide 1
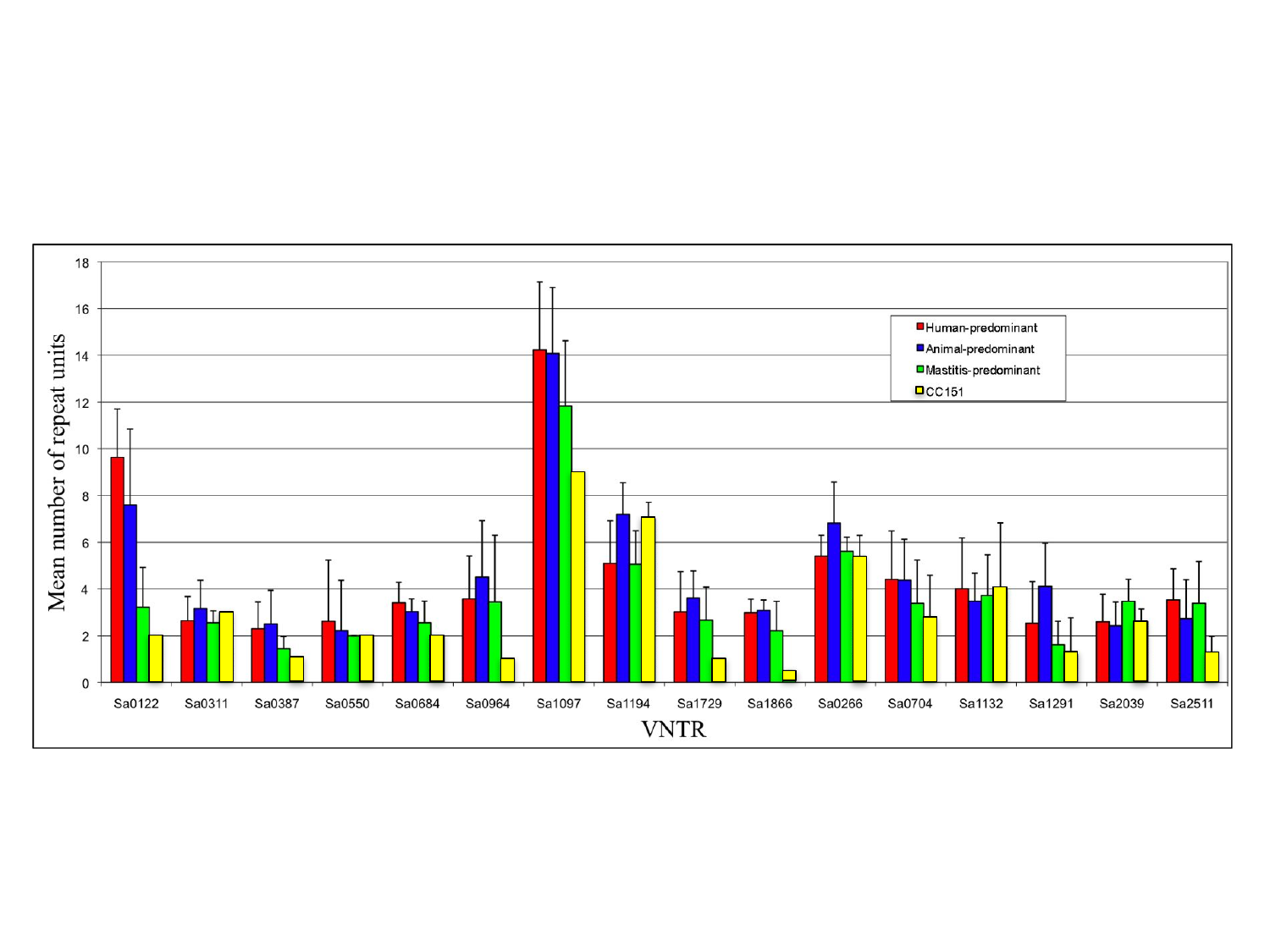

Supplement: Additional file 3: — Mean number of repeat units per locus and for each group (human-predominant, animal-predominant, mammary gland-predominant). For each VNTR, the number of repeat units (mean and standard deviation) are presented for human-, animal- and mammary gland (mastitis)-predominant strains. CC151, belonging to the latter group, is also presented alone. [file 13567_2014_97_MOESM3_ESM.pptx]
